# Supplementary material for: Chromothripsis during telomere crisis is independent of NHEJ, and consistent with a replicative origin
Source: Genome Res. 2019 May;29(5):737–49. doi: 10.1101/gr.240705.118 (PMC6499312; doi:10.1101/gr.240705.118)
Supplement: Supplemental Material [file supp_gr.240705.118_Supplemental_file_1.zip › contigs/annotated_contigs/DB115/contig.2.DB115_length_588_mean_cov_7.84013605442.docx]

**DB115_length_588_mean_cov_7.84013605442**

```
CCTAACACTTTGGGACGTTGAGGCTGGAGAATCACTTGGGCCCAGAAGTTCAAGACCACCCTGGGCAACATAGTGAGACTCCTATCTCT
 >chr3:65970201-65970493 - E=8e-157
AAAAAAAAAAAAAAGAAAAAAAAATTAGCAAGGCATGGTGGTGCATGCCTGTGGTCCCAGCTACTCCCGAGAGTAAGGCTGAAGTGGGA

GGATCACTTAAGCTGGGGAGGCTGAAGCTGCAGTGAGTGGTGATCGTAACACTGTACTCCAGCCTGGGCGATAGAGTGAGACCCTGTCA

CACACACACACACACACACA|CA|TCAGTCACAAAGGGCTTCAAATGTAGATTTTTGGCACTGGTGCCTACTGAGCTTAGATATACAGT
                     >chr3:65968179-65968480 - E=2e-169
CACTATAATGGTGACTGAAATGACAATACAATCATTACGATGTTACACATGAGCCCTATTACCAGACCACACACTCTACAATACAAGAC

ACTGATAATTAATATTTTTTATGAGACCATAATTGTCTCGTAATGGCAGTCACTCCACGTGGCATATTATGTAGGCATTAAAAGCATTG

ATTTATTAGGTATTGATATTTAAAATATACCATCATTTTTACAGCTGTATTCTGGC
```
